# Blood vessel hyperpermeability and pathophysiology in human tumour xenograft models of breast cancer: a comparison of ectopic and orthotopic tumours

**DOI:** 10.1186/1471-2407-12-579

**Published:** 2012-12-05

**Authors:** Karyn S Ho, Peter C Poon, Shawn C Owen, Molly S Shoichet

**Affiliations:** 1 Department of Chemical Engineering & Applied Chemistry, 200 College Street, Toronto, ON, M5S 3E5, Canada; 2 Institute of Biomaterials & Biomedical Engineering, Terrence Donnelly Centre for Cellular and Biomolecular Research, University of Toronto, Room 514 – 160 College Street, Toronto, ON, M5S 3E1, Canada; 3 Department of Chemistry, University of Toronto, 80 St. George Street, Toronto, ON, M5S 3H6, Canada

**Keywords:** Tumour xenograft models, Orthotopic transplantation, Ectopic transplantation, Enhanced permeability and retention, Breast cancer, Blood vessel hyperpermeability, Nanomedicine, Targeting

## Abstract

**Background:**

Human tumour xenografts in immune compromised mice are widely used as cancer models because they are easy to reproduce and simple to use in a variety of pre-clinical assessments. Developments in nanomedicine have led to the use of tumour xenografts in testing nanoscale delivery devices, such as nanoparticles and polymer-drug conjugates, for targeting and efficacy via the enhanced permeability and retention (EPR) effect. For these results to be meaningful, the hyperpermeable vasculature and reduced lymphatic drainage associated with tumour pathophysiology must be replicated in the model. In pre-clinical breast cancer xenograft models, cells are commonly introduced via injection either orthotopically (mammary fat pad, MFP) or ectopically (subcutaneous, SC), and the organ environment experienced by the tumour cells has been shown to influence their behaviour.

**Methods:**

To evaluate xenograft models of breast cancer in the context of EPR, both orthotopic MFP and ectopic SC injections of MDA-MB-231-H2N cells were given to NOD scid gamma (NSG) mice. Animals with matched tumours in two size categories were tested by injection of a high molecular weight dextran as a model nanocarrier. Tumours were collected and sectioned to assess dextran accumulation compared to liver tissue as a positive control. To understand the cellular basis of these observations, tumour sections were also immunostained for endothelial cells, basement membranes, pericytes, and lymphatic vessels.

**Results:**

SC tumours required longer development times to become size matched to MFP tumours, and also presented wide size variability and ulcerated skin lesions 6 weeks after cell injection. The 3 week MFP tumour model demonstrated greater dextran accumulation than the size matched 5 week SC tumour model (for *P* < 0.10). Immunostaining revealed greater vascular density and thinner basement membranes in the MFP tumour model 3 weeks after cell injection. Both the MFP and SC tumours showed evidence of insufficient lymphatic drainage, as many fluid-filled and collagen IV-lined spaces were observed, which likely contain excess interstitial fluid.

**Conclusions:**

Dextran accumulation and immunostaining results suggest that small MFP tumours best replicate the vascular permeability required to observe the EPR effect in vivo. A more predictable growth profile and the absence of ulcerated skin lesions further point to the MFP model as a strong choice for long term treatment studies that initiate after a target tumour size has been reached.

## Background

Pre-clinical development of anti-cancer therapeutics relies on availability of relevant and reproducible in vivo tumour models. Human tumour xenograft models in immunodeficient mice are widely used to assess pharmacokinetics, biodistribution, and treatment efficacy because they are inexpensive and easy to replicate [1]. However, their utility in evaluating potential treatment strategies depends on their capacity to recapitulate human disease conditions.

Progress in nanomedicine seeks to shift distribution of therapeutic compounds to tumour tissue by targeting hyperpermeable tumour vasculature [2,3]. Tumours are restricted in size until they can trigger greater blood vessel density through angiogenesis and blood vessel remodeling [4,5]. Compared to normal tissue, tumour tissue has been demonstrated to be more permissive to extravasation of macromolecules as a result of abnormal blood vessel structure [3]. Moreover, tumour tissue is subject to poor lymphatic drainage, leading to greater retention of material in the extravascular space. These combined phenomena are called enhanced permeability and retention (EPR) and form the basis for improved selectivity of nanoscale drug delivery for solid tumour targeting [2,4,6].

Several pathological features of tumour vasculature lead to its utility in targeting applications. Pathological tumour vessels are dynamic, and can result both from angiogenesis and remodeling of existing vessels [5,7]. Endothelial cells that comprise tumour blood vessels have poor organization, leading to gaps between cells, multiple endothelial cell layers, and unusual tortuosity and branching [8,9]. These openings allow unregulated movement of macromolecules and nanoscale carriers across tumour vessel walls and into the surrounding tissue [10]. In response, the associated basement membrane is also often thickened or absent [9,11]. This apparent dichotomy stems from a dynamic interaction between increased and multilayered collagen deposition in the basement membrane [10,12-14] and increased expression of matrix metalloproteinases (MMPs) that can result in collagen degradation [15]. Further enhancing the aberrant permeability of tumour blood vessels, the pericytes that normally cover and stabilize the outer vessel wall can also be missing or detached, leading to a more immature vessel structure [5]. The absence of these contractile support cells may lead to further increased vessel permeability and weakened control over blood flow [7]. Lymphatic vessels are closely associated with and derived from the blood vessel network. They are responsible for transporting waste out of tissues, but tumours are often deficient in lymphatic drainage, leading to increased accumulation of macromolecular material in tumour tissue [4]. Each of these features contributes to the pathophysiology that enables the EPR effect. Vascular permeability factors, such as bradykinin and nitric oxide, also mediate and enhance vascular hyperpermeability [16]; however, our analysis will be limited to physical vascular defects and their contributions to EPR.

To validate the use of human tumour xenografts in mouse models of breast cancer to investigate tumour targeting via EPR, we studied MDA-MB-231-H2N cells transplanted in NOD scid gamma (NSG) mice and compared two common cell injection sites in the context of EPR permissive pathology. Owing to its simplicity, tumour cells are often introduced ectopically as subcutaneous (SC) injections, regardless of their native tissue type [17-19]. Cells injected orthotopically (eg. breast cancer cells into mammary tissue) are subject to biological cues present in the relevant organ environment [18]. Allowing tumour cells to grow in their orthotopic environment influences growth rate, blood and lymphatic vessel development, metastatic potential, interstitial pressure, and response to therapy [5,18-21]. We hypothesized that the orthotopic environment may also influence the permeability of the resulting tumour vasculature. Notably, to promote successful tumour engraftment in both locations, our chosen cell line is known to be tumourigenic in the absence of external factors, such as estrogen [22]. Groups of animals were compared as cohorts of matching tumour size because size, and not elapsed time, is a standard prognostic measure used to assess breast cancer stage [23].

Currently, the benefit of using either SC or MFP in xenograft models of breast cancer in assessing targeting through the EPR effect is not well characterized. To investigate vessel permeability, orthotopic and ectopic tumour-bearing NSG mice were given intravenous injections of a fluorescently labeled high molecular weight dextran (FITC-Dextran, 2 MDa, ~80 nm [8]) as a model nanocarrier. After allowing the dextran to circulate, animals were sacrificed, their tumours removed, measured using calipers, and fixed with paraformaldehyde. Tumours were cryosectioned and examined for dextran accumulation. Tissue sections were also immunostained for markers of vascular endothelial cells (CD31), basement membrane (collagen IV), pericytes (alpha smooth muscle actin (αSMA)), and lymphatic vessels (lymphatic vessel endothelial hyaluronan receptor (LYVE-1)).

## Methods

### Materials

All cell culture materials were purchased from Gibco-Invitrogen (Burlington, ON, Canada). MDA-MB-231-H2N cells and NOD scid gamma (NSG) mice were generous gifts from Dr. Robert Kerbel (Sunnybrook Research Institute, Toronto, ON, Canada), which were then maintained or bred in-house. Lysine-fixable dextran-FITC (MW 2 ± 0.2 MDa) was purchased from Invitrogen (Burlington, ON, Canada). Slides and cover slips were purchased from Fisher Scientific (Ottawa, ON, Canada). Primary antibodies were purchased from Abcam (Cambridge, MA, USA) for CD31 (ab28364), LYVE-1 (ab14917), collagen IV (ab19808), and αSMA (ab5694). Immunostaining reagents (rabbit IgG Elite ABC kit, avidin/biotin kit, enzyme substrates, Vectashield mounting medium) were purchased from Vector Labs (Burlington, ON, Canada). Entellan hard mounting medium was purchased from EMD Millipore (Billerica, MA, USA). All other materials were purchased from Sigma-Aldrich (Mississauga, ON, Canada) and used as received unless otherwise noted.

### Cell maintenance and preparation

MDA-MB-231-H2N cells were maintained in RPMI 1640 culture medium, supplemented with 10% heat-inactivated fetal bovine serum (FBS), 50 units/mL penicillin and 50 mg/mL streptomycin under a humidified 5% CO_2_ environment. To prepare cell suspensions for injection, adherent cells were first rinsed with phosphate buffered saline, pH 7.4 (PBS), and then incubated briefly with trypsin-ethylenediamine tetraacetic acid (trypsin-EDTA, 0.25%/0.038%). Once the cells were suspended, enzymatic digestion was inhibited with FBS, and the cells were pelleted and washed 3 times in PBS before resuspension at the desired concentration. Cells were kept on ice prior to injection.

### Tumour xenograft models

The protocols used in these in vivo studies were approved by the University Health Network Animal Care Committee and performed in accordance with current institutional and national regulations. Animals were housed in a 12 h light and 12 h dark cycle with free access to food and water. NSG mice were bred in-house, and 7–9 week old female mice were selected for tumour xenotransplantation.

Mice in all experimental groups were inoculated with 10^6^ MDA-MB-231-H2N cells suspended in 50 μL of sterile PBS. Prior to injection, mice were anaesthetized with isoflurane-oxygen. To form ectopic SC tumours, anaesthetized mice were injected with cells under the skin in the right dorsal flank. To form orthotopic mammary fat pad (MFP) tumours, the surgical area was depilated and swabbed with 70% ethanol and betadine before making an incision in the skin of the lower abdomen to the right of the midline, uncovering the mammary fat pad in the right inguinal region where cells were injected into the fat pad. The incision was then sutured closed and lactated Ringer’s solution and buprenorphine were given post-operatively for recovery and pain management. Solid tumours were allowed to form over a period of 3–5 weeks. Cohorts of tumour-bearing animals were divided into two groups to proceed onwards for testing; the first group was tested once their tumours reached an average diameter along the major axis of 7 mm as measured through the skin using calipers, and the second group tested the following week.

### Dye injections and tissue collection

Once tumours reached their target size, mice were injected with 0.5 mg of FITC-dextran in 200 μL of PBS via intravenous (IV) tail vein injection [8]. After 1 h, animals were sacrificed by CO_2_ asphyxiation and tissue samples (tumour and liver) were collected by dissection; tumour samples were directly measured for diameter along both the major and minor axes (L and W) and thickness (H) using calipers (ellipsoid volume calculated as *π*/*6* × *L* × *W* × *H*[24]), and each sample was placed separately in cassettes and submerged in 4% paraformaldehyde for 24 h at 4°C. Tissue samples were then cryoprotected in 30% sucrose in PBS and stored at 4°C. Tissue samples were cryosectioned in 10 μm sections, and pairs of slices 50 μm apart were mounted onto slides, and stored at −80°C. For fluorescence analysis, slides were rehydrated in PBS and coverslipped using Vectashield mounting medium.

### Immunostaining

Three slides (six tissue sections) from each tumour were selected for each set of stains such that each slide contained sections a minimum of 300 μm away from the previous slide. Thawed slides were hydrated and washed in PBS and incubated with 0.3% H_2_O_2_ in methanol for 20 min before being washed in PBS again and blocked in 1.5% normal goat serum (NGS) in PBS (see Table 1 for details). Avidin and biotin blocking reagents were applied sequentially for 15 min each before incubating with the primary antibody at 4°C overnight (dilutions noted in Table 1). The following day, slides were washed in PBS and incubated with a biotinylated secondary goat anti-rabbit IgG (1:200 dilution as instructed in kit), followed by incubation with avidin-biotinylated enzyme complex (ABC reagent) (times noted in Table 1). Rinsed sections were then developed using 3,3’-diaminobenzidine (DAB) enzyme substrate for 1–10 min (brown product). If applicable, slides were then co-stained by repeating the above procedure beginning at the NGS blocking step, and developed in VIP enzyme substrate for 5–7 min (violet product). All slides were counter stained by applying 0.5% methyl green for 10 min (blue-green nuclear stain), washed in distilled water, dried in 1-butanol, and transferred to xylene before being coverslipped using Entellan hard mounting medium.

**Table 1:** Immunostaining protocol details listed by antigen

	**CD31**	**Collagen IV**	**αSMA**	**LYVE-1**
NGS blocking incubation time	1 h	1 h	1 h	20 min
Primary antibody dilution	1:200	1:1000	1:1000	1:1000
Secondary antibody incubation time	1 h	30 min	30 min	30 min
ABC reagent incubation time	1 h	30 min	30 min	30 min

### Image acquisition and analysis

All fluorescence images (FITC-dextran) were acquired with a fixed exposure time for each channel using an Olympus BX50 with a UPlanSApo 10×/0.40 objective, Photometrics CoolSNAP HQ2 monochrome camera, and motorized stage (Olympus Canada Inc., Richmond Hill, ON, Canada). Images were tiled together using Metamorph, and analyzed using ImageJ. To compensate for blood remaining in tissue after sacrifice, the blood-associated fluorescence intensity was quantified in hepatic sinusoids in liver tissue slices. Pixels matching this intensity were subtracted from the positive pixel count in the subsequent analysis.

Brightfield images (immunostaining) were acquired using an Aperio ScanScope XT (Aperio, Vista, CA, USA) for whole slide scanning at 20× magnification and analyzed using ImageScope Microvessel Analysis. Statistical significance between groups was first tested with Bartlett’s test for equality of variance (*P* < 0.05). Where variances were equivalent, one-way ANOVA was applied, followed by a corrected unpaired t-test; differences are denoted by square bracket symbols connecting the differing groups (*P* < 0.05, unless otherwise noted).

## Results and discussion

### Orthotopic cell transplantation influences tumour growth rate and size variation

**Figure 1:**
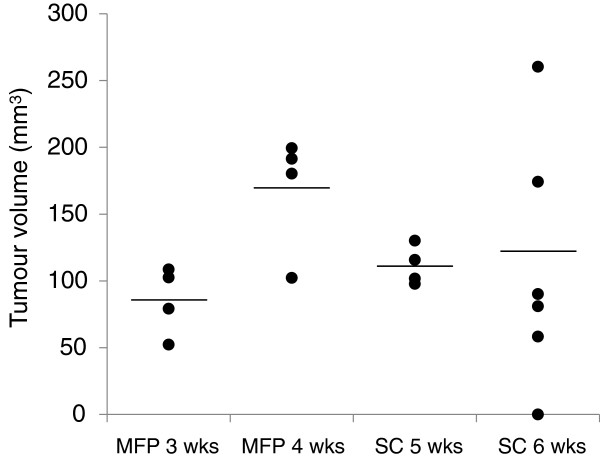
**MFP and SC tumour sizes**. Tumour volumes were calculated based on caliper measurements post-dissection of the major and minor axes and thickness (*n* = 4–6). SC tumours required longer development times to become size matched to MFP tumours. Greater variability was also observed at longer times, particularly in SC tumours, where several animals had smaller tumours than the cohort examined the week before.

Tumour size of human tumour xenograft models grown in mice both orthotopically (MFP) and ectopically (SC) was monitored weekly through the skin in live animals using calipers. Following cell injection, MFP tumours reached a target size of 7 mm in diameter across the major axis by 3 weeks post-injection whereas SC tumours took an additional 2 weeks to reach this size. Differences in growth rate were expected, as each injection site provides a different microenvironment. Cohorts of animals were selected based on tumour size matching instead of development time because size is one of three standard measurements that determines breast cancer prognosis [23]. After resection, tumours were measured directly using calipers and the volumes were calculated based on measurements of the major and minor axes and thickness (Figure 1). The difference in time needed to achieve size matched populations for MFP and SC tumour models suggests that the organ environment influences the growth rate of xenografted cells.

To investigate effects associated with tumour size, 4 animals from each tumour type were randomly selected for dextran injection and tumour resection once the 7 mm major axis diameter was reached, with the remaining animals evaluated the following week. The 7 mm target size was selected to allow adequate vascular pathology to develop, as neovascularization of the tumour is most pronounced over serveral days immediately after a palpable mass (20 mm^3^) has formed [25]. Notably, the week after this target size was reached, tumour size variability increased in both tumour sites (*P* < 0.05 by Bartlett’s test of equality of variances). Unexpectedly, several tumours in the SC group at 6 weeks post-cell injection were smaller than those observed the previous week (Figure 1). Additionally, several replicates were of similar size or larger size, resulting in a broad size distribution of the resulting tumours. In this group, 4 out of 6 animals developed hard fibrotic tissue leading to an ulcerated skin lesion by this time (an indication for humane sacrifice). These lesions, which made these animals unsuitable for further study beyond this time, were not observed in any other group. MFP tumours were grown from cells injected directly into the centre of the MFP, surrounding transplanted cells with endogenous support cells and forming a biological barrier against contact with the skin, which may have prevented ulceration. These injected cells also had access to the pre-existing vascular network and biological signaling molecules present in the MFP. Overall, the MFP tumours were more consistent in size than the SC tumours. Given that the cell line (MDA-MB-231-H2N) and mouse strain (NSG) that we selected are well-established as highly permissive to tumour xenografts [22,26], we expect trends to be similar using other combinations of cells and mice. However, it is possible that other systems will behave differently. Given the large variability in the 6 week SC tumour group, these samples were not further analyzed. Instead, 5 week SC tumours were compared with 3 and 4 week MFP tumours, which were similar in size.

### MFP tumours exceed SC tumours in model nanocarrier accumulation

Prior to sacrifice, a high molecular weight FITC-dextran, used as a model nanocarrier, was injected to assess blood vessel permeability. Data were normalized to liver tissue collected as a positive control: liver endothelial cells have natural fenestrations (123 ± 24 nm diameter) [27] for transfer of substrates from the blood to hepatocytes, making the liver an ideal organ for observing nanocarrier uptake. In mice, blood flow through the liver is also estimated at 23% of cardiac output, making it one of the best perfused organs on a per gram basis [28].

**Figure 2:**
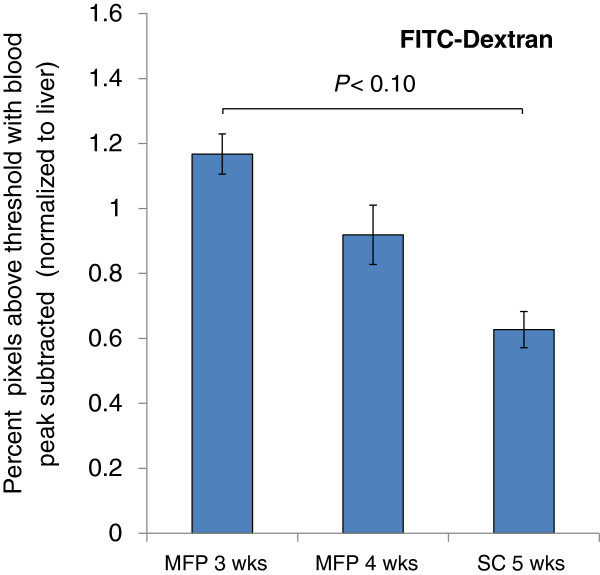
**FITC-Dextran accumulation in tumour tissue normalized to liver tissue control.** High molecular weight dextran (2 MDa, ~80 nm) was injected IV into tumour animals as a model nanocarrier and allowed to distribute prior to sacrifice. 3 week old MFP tumours showed higher accumulation of the nanocarrier than 5 week old SC tumours at a 90% confidence interval. All data are shown as the mean of *n* = 4 animals ± SD. Lines connecting bars denote statistical significance, *P* < 0.10.

Based on fluorescence images of tissue sections, relatively poor dextran uptake was observed in tumour tissue compared to liver tissue across all groups. A threshold was defined to exclude background signal detected in blank tumour and liver tissue and the remaining areas, representing levels above this threshold, were quantified. Less than 1% of the positive signal area observed in the liver control was observed in tumour slices (Figure 2). This can partially be explained by relatively low blood flow through tumour tissue, which has previously been reported to be up to 5-fold lower than in liver [29]. The remaining discrepancy between the dextran accumulation between tumour and liver samples suggests that the model tumour vasculature was less permissive to dextran uptake than the fenestrated liver endothelium, and/or that the lymphatic drainage in the model tumour prevented stable dextran accumulation. Interestingly, dextran uptake in 3 week old MFP tumours was higher than size matched 5 week old SC tumours at 90% confidence (*P* = 0.08 by one-way ANOVA), suggesting that the orthotopic MFP environment encouraged EPR permissive vasculature and/or lymphovasculature.

### Elements of tumour vascular pathophysiology observed in tumour models

**Figure 3:**
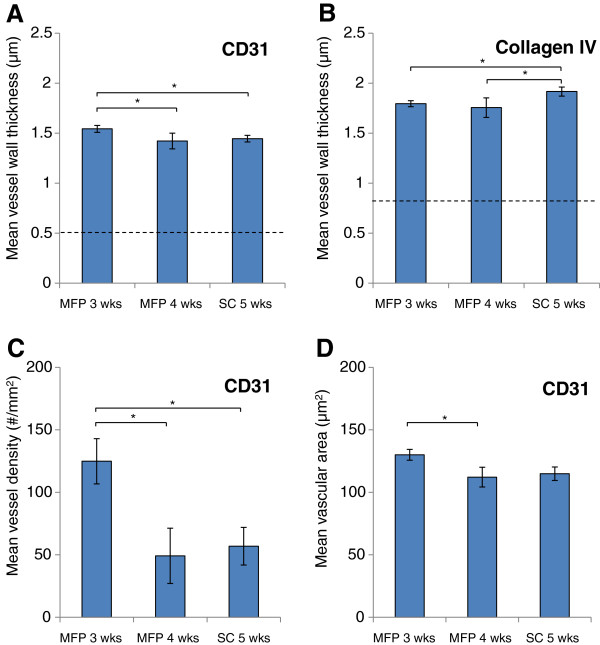
**CD31 and collagen IV immunostaining.** Mean blood vessel wall thickness visualized through **A** CD31 (endothelial cells) and **B** collagen IV (basement membrane). Both are abnormally thick as compared to healthy liver control tissue, which is denoted by the dashed line. **C** shows that mean blood vessel density assayed using CD31 staining is greatest in 3 week old MFP tumours. **D** indicates mean vascular area as a measure of blood vessel size and capacity. Their small size categorizes them as microvasculature. All data are shown as the mean of *n* = 4 animals ± SD. Starred lines connecting bars denote statistical significance, *P* < 0.05.

To better understand the underlying vascular pathophysiology present in both tumour models, tumour slices were immunostained to provide information on the blood and lymphatic vessels present. Tissue was stained for CD31, an endothelial cell marker, to locate and characterize blood vessels. In normal blood vessels, an intact monolayer of endothelials cells is expected, whereas hyperpermeable tumour blood vessels are characterized by multiple layers of discontinuous endothelial cells that may sprout outwards or project into the vessel lumen [10,13]. The CD31 staining revealed greater vessel wall thickness across all groups when compared to liver tissue (represented by a dashed line) which was used as a healthy tissue control (Figure 3A). This observation suggests that blood vessels present in all models, whether they are existing vessels that have been remodeled or newly formed vessels, have the abnormal multi-layered endothelial cell structure associated with solid tumours. The vessel thickness was highest in the 3 week old MFP tumours, indicating a greater level of endothelial cell disorganization in this group. It is possible that this led to the increased permeability observed in the 3 week MFP tumours using a relatively large model nanocarrier (~80 nm), an effect that is more pronounced in other studies utilizing models such as albumin (~7 nm) [30,31].

Separate sections were also co-stained for collagen IV to visualize the thickness of the associated basement membrane. The basement membrane forms a physical barrier that inhibits transport of high molecular weight materials across blood vessel walls [15,32]. In tumour pathophysiology, opposing phenomena have been observed: the basement membrane can thicken, thin, or even be absent. In the MFP and SC tumour models, the basement membrane was thickened compared to healthy liver blood vessels (Figure 3B). This observation is consistent with the xenografted MDA-MB-231-H2N cell line being poorly invasive like its parental line, MDA-MB-231 [33]. Conversely, a more metastatic cell line is often capable of using MMPs to degrade the basement membrane to enable cell migration through neighbouring blood vessels [33]. The 5 week old SC tumours were observed to have the highest basement membrane thickness, indicating the greatest mass transport barrier against nanocarrier delivery.

CD31 staining also revealed differences in vascular density, with the 3 week old MFP tumours having a significantly greater vessel density than the other groups (Figure 3C). The decrease in vascular density from 3 weeks to 4 weeks in the MFP model suggests that the tumour cell growth may be too rapid for the corresponding new blood vessels to form. The thick basement membranes observed in the tumour tissue may also contribute to this deficiency as the basement membrane must be degraded before vascular branching can occur [15]. Although the 3 week old MFP and 5 week old SC tumours were size matched, the MFP model had greater blood vessel density, which may be attributed to greater vascular density in the MFP. Together these observations suggest that remodeling blood vessels already present in the transplantation site are important in establishing relevant tumour vasculature. The relatively poor vascular density in SC tumours may also explain the poor engraftment after 6 weeks, as a lack of blood flow may inhibit further growth and lead to necrosis.

The mean vascular area was also quantified, giving an indication of the size, and therefore the capacity of the blood vessels present in each tumour type. The vascular area in 3 week old MFP tumours was significantly higher than the 4 week old MFP tumours (Figure 3D), indicating that in addition to decreasing vessel density with increasing tumour size, there is on average a lower capacity for blood in the vessels present. Having a greater density and capacity for blood perfusion enhances the likelihood for delivery of materials to the 3 week old MFP tumours through systemic circulation.

At the same time, all of the evaluated models are likely underperfused as their small size categorizes them as microvasculature [34]. This low overall capacity for blood flow impacts their utility in assessing nanocarrier accumulation via EPR, and likely results in regions of hypoxia and heterogeneous drug distribution.

**Figure 4:**
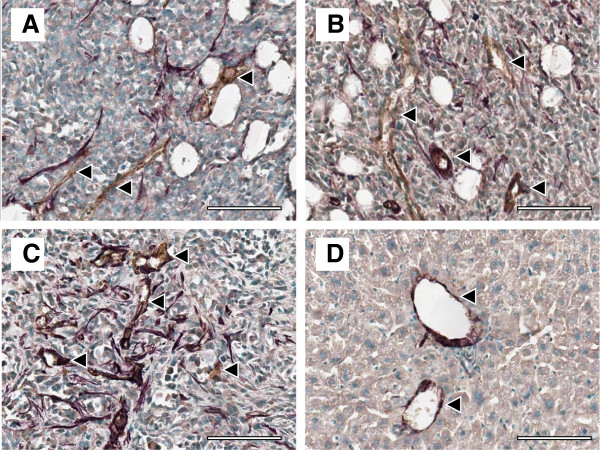
**CD31 and αSMA co-staining.** Representative images of pericytes (αSMA, violet) that are not associated with blood vessels (CD31, brown) in: **A** 3 week MFP, **B** 4 week MFP, and **C** 5 week SC tumours. Several blood vessels are highlighted with black arrows; blue staining represents cell nuclei. **D** shows that pericytes are exclusively associated with blood vessels in healthy liver control tissue. Scale bars represent 200 μm.

CD31 was also co-stained with αSMA to visualize differences in pericyte association with blood vessels. Pericytes are important blood vessel support cells that help to regulate blood flow and vessel permeability, but are often detached in tumour pathophysiology. The observed staining patterns suggest that this was the case across all tumour models (Figure 4A-C). Pericytes (violet) were distributed throughout tumour tissue instead of associating exclusively with blood vessels (brown) and forming uniform layers around the endothelial cell layer, as observed in healthy liver tissue (Figure 4D).

**Figure 5:**
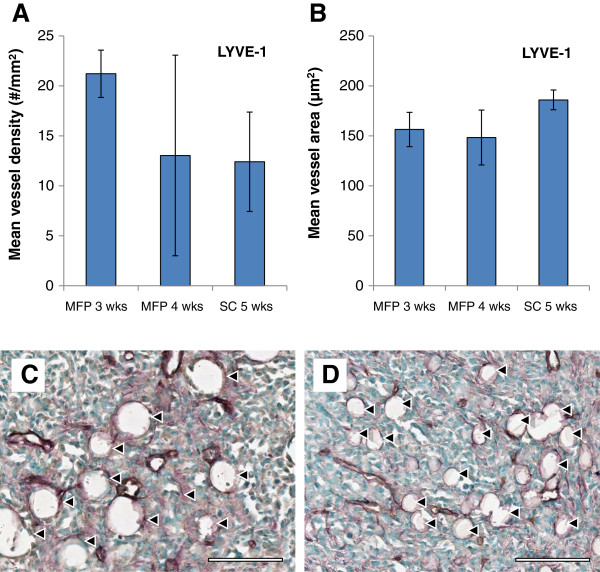
**LYVE-1 immunostaining. A** shows mean lymphatic vessel density, and **B** shows mean vessel area, both of which are indicators of lymphovascular capacity. Both measures were found to have unequal variance between groups, and therefore although the groups were not equivalent, ANOVA could not be used to verify their differences. While 3 week old MFP tumours had the highest mean lymphatic vessel density, 5 week old SC tumours had greater mean vessel size, both of which contribute to overall lymphatic drainage capacity. All data are shown as the mean of *n* = 4 animals ± SD. Representative images of fluid-filled spaces lined with collagen (violet) but not with endothelial cells (negative for CD31, brown)are shown in: **C** 3 week MFP and **D** 5 week SC tumours. Several of these spaces, which indicate lymphedema, are highlighted with black arrows; blue staining represents cell nuclei. Scale bars represent 200 μm.

LYVE-1 staining was used to detect lymphatic vessels in tumour tissue. Lymphatic vessels provide a network to drain protein rich interstitial fluid back into circulation. By the nature of their function, these vessels are porous to allow macromolecules to be transported [35], and therefore nanocarrier accumulation in tumour tissue may increase when their expression is impaired. Mouse models of lymphatic impairment can be generated by surgically ablating lymphatic vessels in the tail, resulting in lymphedema. In these models, the surrounding tissue attempts to restore homeostasis by generating new lymphatic vessels and dilating the remaining lymphatic vessels, suggesting that both density and diameter impact drainage capacity [36]. LYVE-1 stained sections were used to quantify lymphatic vessel size and density (Figure 5A-B). Both of these measures gave different variances between groups (*P* < 0.05 by Bartlett’s test of equality of variances) meaning that the groups tested were not equivalent. While the mean lymphatic vessel density was highest in the 3 week old MFP tumours, the 5 week old SC tumours demonstrated the highest mean lymphatic vessel area. These factors counterbalance one another, as density and capacity each contribute to overall drainage.

There is evidence that both the MFP and SC tumour models yielded poor lymphatic drainage compared to healthy tissue. Accumulation of interstitial fluid in cases of lymphedema has been shown to lead to the deposition of collagen [37]. Visual examination of the tumour slices revealed a high density of collagen IV-lined spaces that were CD31 negative, which likely represent fluid-filled cavities in the tumour tissue (Figure 5C-D). These likely contain excess interstitial fluid resulting from a combination of increased vascular permeability and deficient lymphatic drainage.

Taken together, the data gathered through CD31 and collagen IV immunostaining suggest that, of the models tested, the 3 week MFP tumour best replicates the vascular permeability required to observe the EPR effect in vivo. However, the blood vessels visualized are sparse and small, contributing to low accumulation of the model nanocarrier used in this study. Both MFP and SC tumours showed evidence of excess interstitial fluid accumulation, suggesting poor lymphatic drainage in both models. While MFP tumours demonstrated greater lymphatic vessel density, SC tumours had greater lymphatic vessel size, both of which contribute to drainage, making it difficult to easily differentiate the two models in terms of drainage capacity. MFP tumours demonstrated greater utility for long-term treatment studies, as their growth is more consistent at large tumour sizes, and no skin ulcerations were observed.

## Conclusions

This study provides insight into the vascular properties of human tumour xenograft models of breast cancer in both MFP (orthotopic) and SC (ectopic) environments, two common pre-clinical models. When both animal models were challenged with a high molecular weight dextran as a model nanocarrier, there was higher accumulation in MFP tumours 3 weeks after cell injection. Further adding to the evidence that MFP tumour vasculature has greater permeability to macromolecules – a pathological feature relevant to nanocarrier accumulation via EPR – CD31 and collagen IV immunostaining revealed greater vascular density and size, as well as thinner basement membranes, in MFP tumours collected 3 weeks after cell injection. Both models demonstrated poor dextran accumulation compared to the liver as a positive control, suggesting that although several pathological features were observed, low vascular density and small blood vessel size led to relatively poor tumour perfusion. Both the MFP and SC tumour models showed evidence of poor lymphatic drainage, as several CD31 negative and collagen IV-lined fluid-filled cavities were observed. The MFP environment offered several practical benefits, including shorter development times to reach a target tumour size, more consistent growth profiles, and the absence of ulcerated skin lesions observed in SC tumour animals.

## Abbreviations

α-SMA: Alpha smooth muscle actin; DAB: 3,3’-diaminobenzidine; EPR: Enhanced permeability and retention; FBS: Fetal bovine serum; LYVE-1: Lymphatic vessel endothelial hyaluronan receptor; MFP: Mammary fat pad; MMP: Matrix metalloproteinase; NGS: Normal goat serum; NSG mice: NOD scid gamma mice; PBS: Phosphate buffered saline, pH 7.4; SC: Subcutaneous.

## Competing interests

The authors declare that they have no competing interests.

## Authors’ contributions

KSH designed the study and protocols, performed animal experiments, immunostained tissue, collected images, maintained and prepared cells for transplantation, executed the data analysis, and prepared the manuscript. PP was responsible for the breeding the mouse colony, performing cell injections, monitoring tumour growth, and assisted in designing protocols, performing the animal experiments, immunostaining tissue, and collecting images. SCO participated in designing the study and protocols, and assisted in performing SC cell injections. MSS participated in study design and was involved in writing the manuscript. All authors read and approved the final manuscript.

## Pre-publication history

The pre-publication history for this paper can be accessed here:

http://www.biomedcentral.com/1471-2407/12/579/prepub
